# Improving door-to-reperfusion time in acute ischemic stroke during the COVID-19 pandemic: experience from a public comprehensive stroke center in Brazil

**DOI:** 10.3389/fneur.2023.1155931

**Published:** 2023-07-10

**Authors:** Marcelo Klu, Ana Claudia de Souza, Leonardo Augusto Carbonera, Thais Leite Secchi, Arthur Pille, Marcio Rodrigues, Rosane Brondani, Andrea Garcia de Almeida, Angélica Dal Pizzol, Daniel Monte Freire Camelo, Gabriel Paulo Mantovani, Carolina Oldoni, Marcelo Somma Tessari, Luiz Antonio Nasi, Sheila Cristina Ouriques Martins

**Affiliations:** ^1^Emergency Department, Hospital de Clínicas de Porto Alegre, Universidade Federal do Rio Grande do Sul, Porto Alegre, Brazil; ^2^Neurology Department, Hospital de Clínicas de Porto Alegre, Universidade Federal do Rio Grande do Sul, Porto Alegre, Brazil; ^3^Neurology Department, Hospital Moinhos de Vento, Porto Alegre, Brazil

**Keywords:** stroke, thrombolysis, COVID-19, quality of care, door to needle time

## Abstract

**Background:**

The global COVID-19 pandemic has had a devastating effect on global health, resulting in a strain on healthcare services worldwide. The faster a patient with acute ischemic stroke (AIS) receives reperfusion treatment, the greater the odds of a good functional outcome. To maintain the time-dependent processes in acute stroke care, strategies to reorganize infrastructure and optimize human and medical resources were needed.

**Methods:**

Data from AIS patients who received thrombolytic therapy were prospectively assessed in the emergency department (ED) of Hospital de Clínicas de Porto Alegre from 2019 to 2021. Treatment times for each stage were measured, and the reasons for a delay in receiving thrombolytic therapy were evaluated.

**Results:**

A total of 256 patients received thrombolytic therapy during this period. Patients who arrived by the emergency medical service (EMS) had a lower median door-to-needle time (DNT). In the multivariable analysis, the independent predictors of DNT >60 min were previous atrial fibrillation (OR 7) and receiving thrombolysis in the ED (OR 9). The majority of patients had more than one reason for treatment delay. The main reasons were as follows: delay in starting the CT scan, delay in the decision-making process after the CT scan, and delay in reducing blood pressure. Several actions were implemented during the study period. The most important factor that contributed to a decrease in DNT was starting the bolus and continuous infusion of tPA on the CT scan table (decreased the median DNT from 74 to 52, DNT ≤ 60 min in 67% of patients treated at radiology service vs. 24% of patients treated in the ED). The DNT decreased from 78 min to 66 min in 2020 and 57 min in 2021 (*p* = 0.01).

**Conclusion:**

Acute stroke care continued to be a priority despite the COVID-19 pandemic. The implementation of a thrombolytic bolus and the start of continuous infusion on the CT scan table was the main factor that contributed to the reduction of DNT. Continuous monitoring of service times is essential for improving the quality of the stroke center and achieving better functional outcomes for patients.

## Introduction

Stroke is the second leading cause of death and the main cause of disability in Brazil and worldwide (1, 2). Since the proven benefit of thrombolysis for reperfusion in acute ischemic stroke (AIS) in 1995 (3), hospitals have implemented protocols, workflows, and training for physicians and nurses in all stages of the process, allowing for rapid assessment and treatment. Intravenous thrombolysis (IVT) with a recombinant tissue plasminogen activator (rtPA) has proven benefits up to 4.5 h from symptoms onset (4), but its effectiveness decreases over time.

Therefore, stroke teams worldwide have been making efforts to initiate reperfusion treatment as soon as possible after the patient arrives in the emergency department (ED). Several studies have attempted to identify the reasons for treatment delay and create strategies to reduce the time between arrival at the ED and the start of intravenous thrombolysis (door-to-needle time, DNT) (5–8). It has been well-demonstrated that the DNT can be much lower than the goal of < 60 min, and there is currently a recommendation that at least 50% of treatments should be performed in < 45 min (9).

In-hospital treatment delay may occur during any stage of acute stroke treatment, including patient triage, medical assessment, neuroimaging, blood sample collection and analysis, obtaining consent, treatment of high blood pressure, and the decision-making process (6, 10, 11). The implementation of protocols with a well-trained team and the division of duties in each stage may reduce treatment delay. Monitoring treatment times supports identifying the reasons for the delay and allows for structuring the service for better patient outcomes (12, 13).

The global COVID-19 pandemic has had a major impact on all aspects of emergency stroke healthcare, including the pre-hospital care system and in-hospital workflow. There has been a significant reduction in the number of stroke admissions and stroke patients treated with reperfusion therapy worldwide (14–17). Brazil recorded its first COVID-19 patient on 26 February 2020 and experienced rapid spread of the infection. Stroke team members had to make substantial adaptations to current stroke care protocols in emergency rooms due to the institution of infection control measures. Some centers have reported increased door-to-needle times during the COVID-19 pandemic, while others have noted no change (14, 17, 18).

The aim of this study is to assess rates of IVT treatment, evaluate the reasons for the delay in each stage of IVT treatment in a public stroke center, and propose and implement strategies to reduce treatment times based on the results obtained.

## Methods

### Hospital structure

Hospital de Clínicas de Porto Alegre (HCPA) is a public university hospital located in Southern Brazil that has had a well-structured acute stroke unit (ASU) in the ED since 2006 (19) and a comprehensive stroke unit (CSU) since 2012. It was the first hospital in Brazil to be licensed as a stroke center in 2012 by the Ministry of Health. Since 2013, after the implementation of the National Stroke Policy, the number of stroke centers in the region has increased from 2 to 16. This also included engagement with emergency mobile care service, the well-organized public pre-hospital Emergency Medical System (EMS). As a result of the aforementioned strategies, the overall number of thrombolysed patients has increased in the entire region, avoiding the burden on a single-stroke center. Since then, the number of thrombolysed patients in this hospital has remained stable between 70 and 80 patients per year.

The stroke center is staffed by a trained multidisciplinary stroke team, which includes four stroke neurologists who provide supervision and support to neurology residents. This occurs on-site during the day and by telemedicine during the night. Neurology residents provide on-site coverage during the day and on-call coverage from 6 p.m. to 8 a.m. The stroke team is available 24 h a day, 7 days a week, on a rotating schedule to support all stroke cases from arrival in the ED to the hospital discharge. Additionally, patients are followed up by the stroke team at the stroke outpatient clinic.

The hospital maintains a prospective data registry for all consecutive stroke patients to monitor the quality of stroke care. The diagnosis of AIS was confirmed by a computed tomography (CT) scan on admission. Acute stroke treatment protocols of the Brazilian Stroke Society/Brazilian Academy of Neurology (20, 21) were followed, which are in accordance with the American Stroke Association Guidelines (9).

### Patient workflow and treatment protocols

The in-hospital workflow for stroke patients begins upon their arrival at the ED. At this moment, the stopwatch is started. The use of the stopwatch has been part of the stroke protocol since the hospital was recognized as a stroke center. Patients can arrive in two different ways: approximately 70% of the patients are brought by EMS (SAMU), while the remaining patients arrive by their own means (family vehicle, taxi, or bus). SAMU is trained in pre-hospital stroke care and operates within a network that allocates AIS patients among the three public stroke centers in the city of Porto Alegre since 2008 and among the 16 stroke centers licensed by the Ministry of Health since 2013.

SAMU notifies the destination hospital in advance that an IVT candidate is being transported. The patient is evaluated by a triage nurse, who then immediately directs the SAMU team to the ASU in the ED. The administrative receptionist opens the patient's file to allow access to the patient's medical records, and until this process is completed (which takes up to 10 min), no tests can be ordered. When patients arrive by their own means, they wait for triage and risk classification with other patients. Once a possible AIS is identified by the triage nurse, the patient is immediately transported to the ASU.

At the ASU, nurses and physicians simultaneously begin the patient's assessment and call the neurologist over the phone. Blood pressure and capillary glycemia are measured, and two venous accesses are punctured. Since 2018, all the supplies and medication needed to evaluate and treat patients with AIS (including blood sample tubes, blood pressure-lowering agents, and rtPA) have been stored in a stroke box located in the ASU.

All neurologists and residents are certified in the use of the National Institute of Health Stroke Scale (NIHSS). Acute stroke patients are assessed by neurology residents in the ED, where the severity of the neurological deficit on admission is evaluated using the NIHSS. The patient is monitored and taken, along with the stroke box, to the neuroimaging acquisition room, which is 225 m and 4 min 15 s from the ED. The elevator is still used on this route as the radiology service is on the second floor. Whenever the stroke neurologist is in the hospital, cases are discussed face-to-face with residents, and the images are assessed on a PACS workstation. Otherwise, all relevant clinical information and DICOM images are shared in real-time with the entire stroke team *via* a smartphone application (Join^©^ App, Allm Inc. Tokyo, Japan), which is locally validated for use as a telemedicine device for stroke treatment assessment (22). After the neuroimaging acquisition, the patient returns to the ASU, and the stroke neurologist reviews the clinical and imaging data in the Join^©^ App, deciding on reperfusion therapy with IVT or conservative treatment.

The times for treatment were collected from the medical records, including the time of arrival at the ED, time of risk classification (time of triage), time of the neurologist's call, time of the neurologist's arrival, time of the tPA bolus (registered by the nurse in the ED or on the smartphone application when the tPA bolus and infusion started on the CT scan table), and the CT scan time, which is collected in the first image. Reasons for treatment delay were collected from the medical records, stroke database, and Join^©^ App.

### Barriers identified prior to this study

Some factors that may possibly delay the patient's treatment were previously identified. (1) The neurology resident is on call from 6 p.m. to 8 a.m. and needs to go to the hospital to assess the patient. (2) The time required to open the patient's medical record delays the performance of laboratory tests and neuroimaging. (3) Blood sample collection is delayed whenever the lab collector is not available, resulting in a delay in neuroimaging acquisition; (4) The distance between the ED and the radiology service delays the neuroimaging acquisition and transportation back to the ED delays the start of IVT, when applicable; (5) Acquisition of CT angiography before transporting the patient back to the ED and acquisition of brain magnetic resonance imaging (MRI) for patients with unknown onset of symptoms also delay the initiation of treatment—MRI is not always available, and even the rapid protocol takes longer than CT scan. (6) The carrier to take the patient to radiology service was not always available; however, this issue was resolved in 2017 with the decision that the nursing staff and the emergency physician (or the neurologist) are allowed to take the patient to radiology, without having to wait for the carrier.

### Influence of the COVID-19 pandemic on the acute stroke care pathway

Many stroke centers around the world have reported a decrease in the number of stroke admissions, intravenous thrombolysis, and mechanical thrombectomy volumes compared to the pre-COVID-19 era. The turning of health staff and hospital resources toward the COVID-19 emergency inevitably led to an important impairment in stroke care worldwide (16, 18, 23). Since the onset of the COVID-19 pandemic, Brazil has adopted unprecedented measures such as social isolation and nationwide lockdown at great economic cost.

Acute stroke protocols, including the adequate screening of symptoms and signs of COVID-19 infection, pathways for acute stroke treatments, and isolation of patients in protected areas, were adjusted according to the Brazilian guideline indications for the management of acute stroke care during the COVID-19 pandemic (18, 23). All stroke patients were tested for COVID-19 infection. Patients with unknown COVID-19 status were evaluated with appropriate personal protective equipment in the ED.

### Study design

We conducted a prospective study in a public university hospital, Hospital de Clínicas de Porto Alegre (HCPA), over a 3-year period (January 2019 to December 2021). During 2019, we evaluated the reasons for the delay, and in 2019 and 2020, we implemented strategies to improve the door-to-reperfusion time. In 2020, the study was affected and therefore changed due to the coronavirus pandemic. Therefore, in 2020 and 2021, we evaluated the effects of the pandemic on the volume of stroke patients, thrombolysis rates, and reperfusion time. We included all consecutive patients with AIS who arrived at the ED and received IVT in the study. We excluded patients who had a stroke in the hospital and patients who received thrombectomy without IVT.

The main objective was to identify specific factors associated with delay in hospital treatment through prospective monitoring of all treatment times, from the patient's arrival in the ED to the start of IVT treatment. Table 1 shows the maximum expected times for each stage. Times above these targets were considered reasons for the delay. DNT medians were analyzed according to the patient's clinical variables, means of transport to the hospital, and in-hospital procedures. We compared patients with DNT of ≤ 60 min with those with more than 60 min. We also described changes to the stroke protocol to improve treatment times during the study and the repercussion of the COVID-19 pandemic on acute stroke care.

**Table 1:** Recommended time targets for stroke care.

**Times**	**Maximum expected time**
Door-to-triage time	5 min
Triage-to-neurologist call (time from triage to the emergency physician call to the neurologist)	15 min
Neurologist call-to-neurologist evaluation	15 min
Door-to-CT	20 min
CT-to-needle time	20 min
Door-to-needle time (DNT)	60 min

Powers (9); Ruff (17).

### Statistical analysis

Categorical variables were presented as proportions and comparisons were made using the χ2 or Fisher exact tests. Continuous variables were shown as a median and interquartile range (IQR) of 25–75%, and the Mann–Whitney U-test was used for comparisons. Comparisons were made according to DTN (≤ 60 min vs. >60 min). A *p*-value below 0.05 was considered to be statistically significant. In addition, multiple logistic regression analysis was performed to evaluate the independent factors in the stroke treatment related to DNT delay. In this study, we included the regression model variables based on results from other studies and on clinical observation and variables that had a *p*-value of < 0.10 in univariate tests. All data were analyzed using SPSS for Windows version 20 (SPSS Inc, Chicago, IL). Informed consent was obtained from all patients or family members. The study protocol was approved by the Ethics Committee of the Hospital de Clínicas de Porto Alegre.

## Results

Between January 2019 and December 2020, 1,739 patients with suspected AIS were evaluated in the ED. Of these, 567 AIS cases were confirmed in 2019 and 753 in 2020, with 143 patients receiving IVT (82 in 2019 and 61 in 2020). In 2021, there were 1,038 suspected AIS cases, 789 confirmed AIS cases, and 113 patients received thrombolytic therapy. Data from 2021 were used for comparison with the main study period, which was 2019 and 2020. The median age was 69 years (ranging from 29 to 99 years), where 49% were men, and 69% of them arrived through SAMU.

Table 2 shows the median DNT according to baseline patient characteristics in 2019 and 2020. Patients with unknown onset of symptoms had slightly longer DNT time but without statistical significance. Patients who underwent an MRI on admission had a higher median DTN time compared to those who had a CT scan (96 min [IQR 85–113] vs. 70 min [55–92], *p* = 0.61). Patients with a previous stroke had a longer median DNT (84 [IQR 64–110] vs. 69 [56–87], *p* = 0.05), while patients who arrived through EMS had a shorter DNT (70 [IQR 57–87] vs. 80 [55–111], *p* = 0.012). Patients who received IVT bolus on the CT scan table had a shorter DNT compared to those treated at the ASU (52 min [IQR 39–71] vs. 74 min [IQR 62–94], *p* < 0.0001).

**Table 2:** Median door-to-needle time according to the baseline characteristics.

	**No (%)**	**Median DNT [IQR]**	**Min-Max**	***p***
**Age** ≥**70**				0.29
No	72 (50)	74 [60–94]	31–165	
Yes	71 (50)	69 [52–92]	23–145	
**Male sex**				0.61
No	73 (51)	69 [56–93]	23–145	
Yes	70 (49)	74 [59–93]	31–165	
**Baseline NIHSS** ≥**15**				0.09
No	106 (74)	69 [55–90]	23–165	
Yes	37 (26)	81 [62–105]	31–140	
**Mechanical thrombectomy**				0.89
No	132 (92)	72 [55–83]	23–165	
Yes	11 (8)	73 [63–87]	43–114	
**Hypertension**				0.59
No	26 (18)	70 [50–93]	33–165	
Yes	117 (82)	73 [59–93]	23–158	
**Diabetes**				0.15
No	97 (68)	73 [61–93]	31–165	
Yes	46 (32)	68 [49–94]	23–158	
**Ischemic heart disease**				0.61
No	120 (86)	72 [56–93]	23–165	
Yes	20 (14)	71 [54–91]	38–107	
**Atrial fibrillation**				0.10
No	111 (78)	68 [54–91]	23–165	
Yes	31 (22)	78 [67–98]	31–128	
**Previous stroke**				0.05
No	107 (75)	69 [56–87]	31–165	
Yes	35 (25)	84 [64–110]	23–158	
**Magnetic resonance in acute stroke**				0.61
No	134 (94)	70 [55–92]	23–165	
Yes	9 (6)	96 [85–113]	56–134	
**Time of arrival**				0.45
7:00 am to 6:59 pm	92 (64)	69.5 [53–93]	23–165	
7:00 pm to 10:59 pm	38 (27)	73 [64–91]	31–132	
11:00 pm to 6:59 am	13 (9)	80 [62–109]	48–158	
**Arrival by EMS**				0.01
No EMS	45 (32)	80 [55–111]	38–165	
EMS (SAMU)	98 (68)	70 [57–87]	23–140	
**Local of thrombolysis**				< 0.0001
At the acute stroke unit (ED)	119 (83)	74 [62–94]	37–163	
At the CT scan room	24 (17)	52 [39–71]	23–108	

EMS, emergency medical service; MRI, magnetic resonance imaging.

Table 3 shows the proportion of patients with DNT ≤ 60 min in the univariate analysis based on baseline characteristics. Patients with diabetes were more likely to be treated within ≤ 60 min (46 vs. 26%). However, patients with AF were treated later, with only 9% receiving treatment within ≤ 60 min compared to 28% receiving treatment after >60 min (*p* = 0.01). Patients treated on the CT scan table were more likely to receive IVT within ≤ 60 min when compared to patients treated in the ED (67 vs. 24%, *p* = 0.0001). Among the 143 thrombolysed patients, only 9 were assessed through brain MRI at admission, and only one was treated in ≤ 60 min. Of the 44 patients treated with IVT in ≤ 60 min, 66% of them arrived by EMS. There was no significant difference in the proportion of patients treated with DNT ≤ 60 min between patients who arrived within 0 to 60, 61 to 120, 121 to 180, or >180 min from the symptom onset (33, 26, 36, and 35%, respectively, *p* = 0.68).

**Table 3:** Proportion of patients with DNT ≤ 60 min according to the baseline characteristics.

	***N***	**DNT > 60 min**	**DNT ≤ 60 min**	***p***
		**99 (69%)**	**44 (31%)**	
**Age** **≥70**	71	44 (48%)	24 (55%)	0.44
**Male sex**	70	50 (51%)	20 (46%)	0.58
**Baseline NIHSS** **≥15**	37	29 (29%)	8 (18%)	0.16
**Mechanical thrombectomy**	11	10 (10%)	1 (2%)	0.17
**Risk factor**				
Diabetes	46	26 (26)	20 (46)	**0.02**
Ischemic heart disease	20	13 (14)	7 (16)	0.71
Atrial fibrillation	31	27 (28)	4 (9)	**0.01**
Previous stroke	35	27 (28)	8 (18)	0.23
**Time of arrival**				
7:00 am to 6:59 pm	92	58 (60)	34 (77)	**0.09**
7:00 pm to 10:59 pm	38	31 (31)	7 (16)	
11:00 pm to 6:59 am	13	10 (10)	3 (7)	
**Arrival by EMS**	98	69 (70)	29 (66)	0.65
**MRI in acute phase**	9	8 (8)	1 (2)	0.28
**Thrombolysis on CT scan**	24	28 (24)	16 (67)	**< 0.0001**

EMS, emergency medical service; MRI, magnetic resonance imaging.

Most patients experienced multiple reasons for treatment delay (Table 4). The most common reason was the delay in starting the IVT after the CT scan, with a CT-to-needle time of >20 min in 90% of the patients. The second most frequent reason for the delay was the patient's return to the emergency room for treatment (77%), followed by the delay in starting the CT scan in radiology service (65%) and performing CT angiography before IVT (46%). Only 8% of patients had no delay in IVT, considering the benchmarks in Table 1. Table 5 shows the main reasons for delay in each patient, defined as the major cause of delay. The main reason was the delay to start the CT scan (22%), which specifically refers to the delay in the radiology service initiating the CT scan, followed by the delay in starting IVT after the CT scan (19%) and the delay in decreasing blood pressure (11%). The delay in triage, in the emergency department, image acquisition in the radiology service, as well as in stabilizing the patient, correspond to the pre-imaging delay, increasing the door-to-CT time. Conversely, delays in the laboratory and delays in the definition and initiation of IVT treatment after the image correspond to the post-imaging delay.

**Table 4:** All reasons for the door-to-needle time delay^*^.

**Reasons for the DNT delay**	**Benchmark**	***N* (%)**
Delay to start the treatment (CT-to-treatment delay)	20 min	129 (90)
Thrombolysis in the emergency room	60 min	91 (77)
Delay to start CT scan	20 min	93 (65)
Perform CTA before IVT	-	66 (46)
Delay in triage	5 min	45 (32)
Delay in reducing blood pressure	-	27 (19)
Lack of information (patient alone)	-	27 (19)
Delay in neurology evaluation	15 min	27 (19)
Delay in emergency physician evaluation	15 min	21 (15)
Unstable patient	-	12 (8)
Perform MRI for thrombolysis selection	-	10 (7)
Delay in the laboratory (anticoagulated patients)	30 min	7 (5)
Delay in the consent form	-	9 (8)
Delay in uploading images in the application	-	5 (4)
Wrong diagnosis in triage	-	5 (4)
Difficult venous access	-	5 (4)
Delay to collect blood exams	-	5 (4)
Delay in triage due to COVID	5 min	2 (1)
Any delay	-	12 (8)

^*^Each patient can have more than one reason for delay.

**Table 5:** Main reasons for the delay in door-to-needle time for each patient^*^.

**Reasons for the DNT delay**	***N* (%)**
Delay to start CT scan	31 (22)
Delay to start the treatment after CT scan	27 (19)
Delay in reducing blood pressure	15 (11)
Delay in the emergency department	11 (8)
Unstable patient	12 (8)
Perform MRI for thrombolysis selection	10 (7)
Delay in the laboratory (anticoagulated patients)	7 (5)
Perform CTA before IVT	6 (4)
Wrong diagnosis in triage	3 (2)
Delay in triage	3 (2)
Difficult venous access	3 (2)
Delay in the consent form	2 (1)
Delay in uploading images in the application	1 (< 1)
No delay	12 (8)

^*^One main reason per patient.

In the multivariate analysis to evaluate independent factors for treatment delay, including age, gender, baseline NIHSS score, AF, arrival by EMS, and the use of MRI in the initial evaluation and treatment in the ED, only AF (OR, 6.8; 95% CI, 1.8 to 26.2) and thrombolysis in the ED (OR, 8.9; 95% CI, 2.9 to 27.5) were independent factors for treatment delay.

### Actions implemented to decrease the DNT

**Figure 1:**
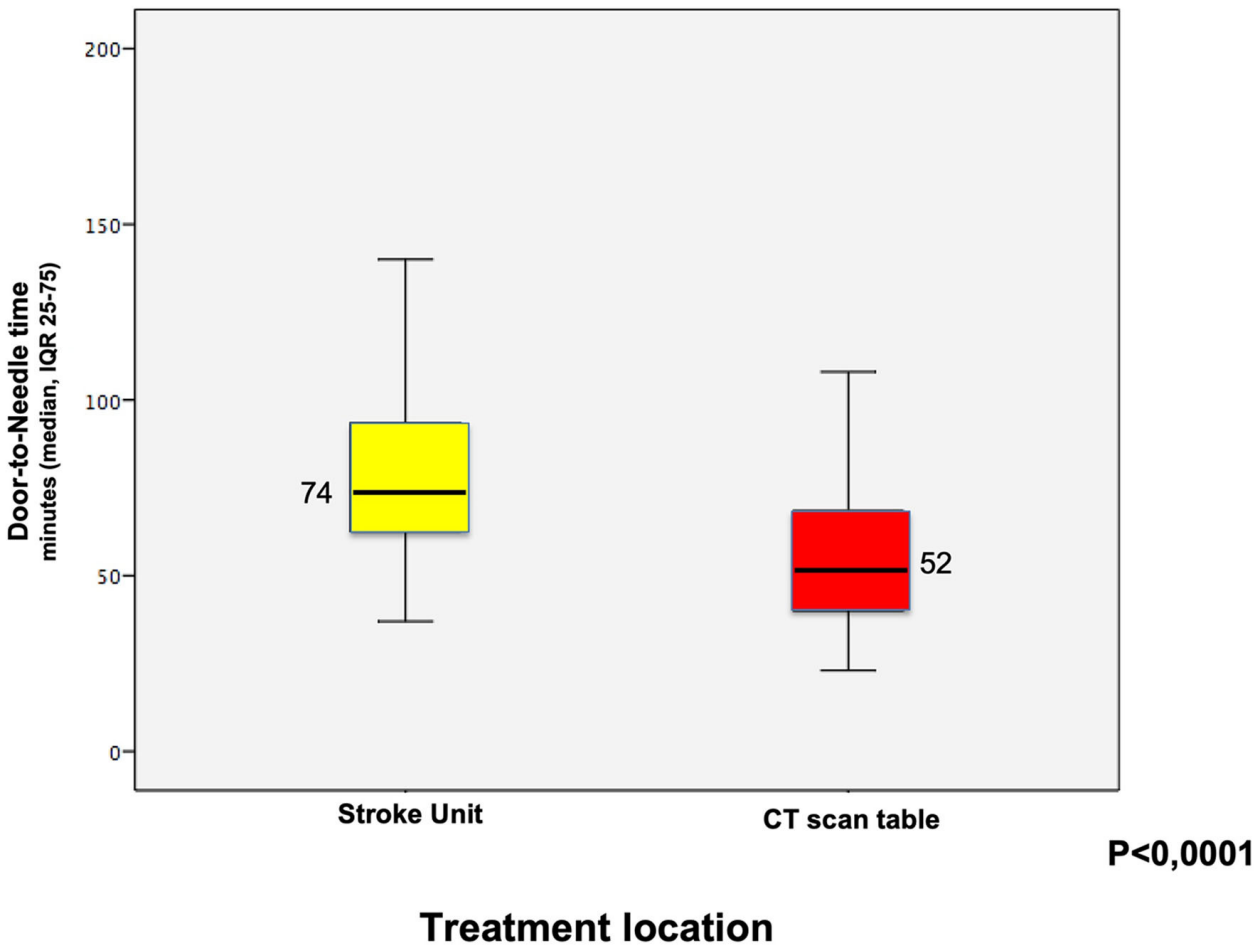
Median times (IQR 25–75) according to the local of thrombolysis.

Specific actions shown in Table 6 were implemented to decrease DNT in addition to the actions that occur every year as stroke campaigns to inform the population and training for the pre-hospital team, emergency staff, neurologists, and radiology team. The main action was to start IVT bolus and continuous infusion on the CT scan table (Figure 1).

**Table 6:** Actions implemented to decrease the DNT during 2019–2020.

**Action**	**Date**	**Result**
Collection of blood tests by nursing staff when the first *abocath* is punctured	January 2019	Decreased the delay in the blood sample collection
Urgent registry of a patient at the emergency arrival	June 2019	Decreased the delay in requesting the exams
Training of the neurology residents to prepare the rtPA in the radiology service	September 2019	The residents started to initiate the thrombolytic treatment in the CT scan table
Training of the neurology residents to a faster blood pressure control	September 2019	Faster BP control
Pack of urgent exams in the electronic system (including CT, CTA, and laboratory exams)	December 2019	Faster transfer of the CT scan

**Figure 2:**
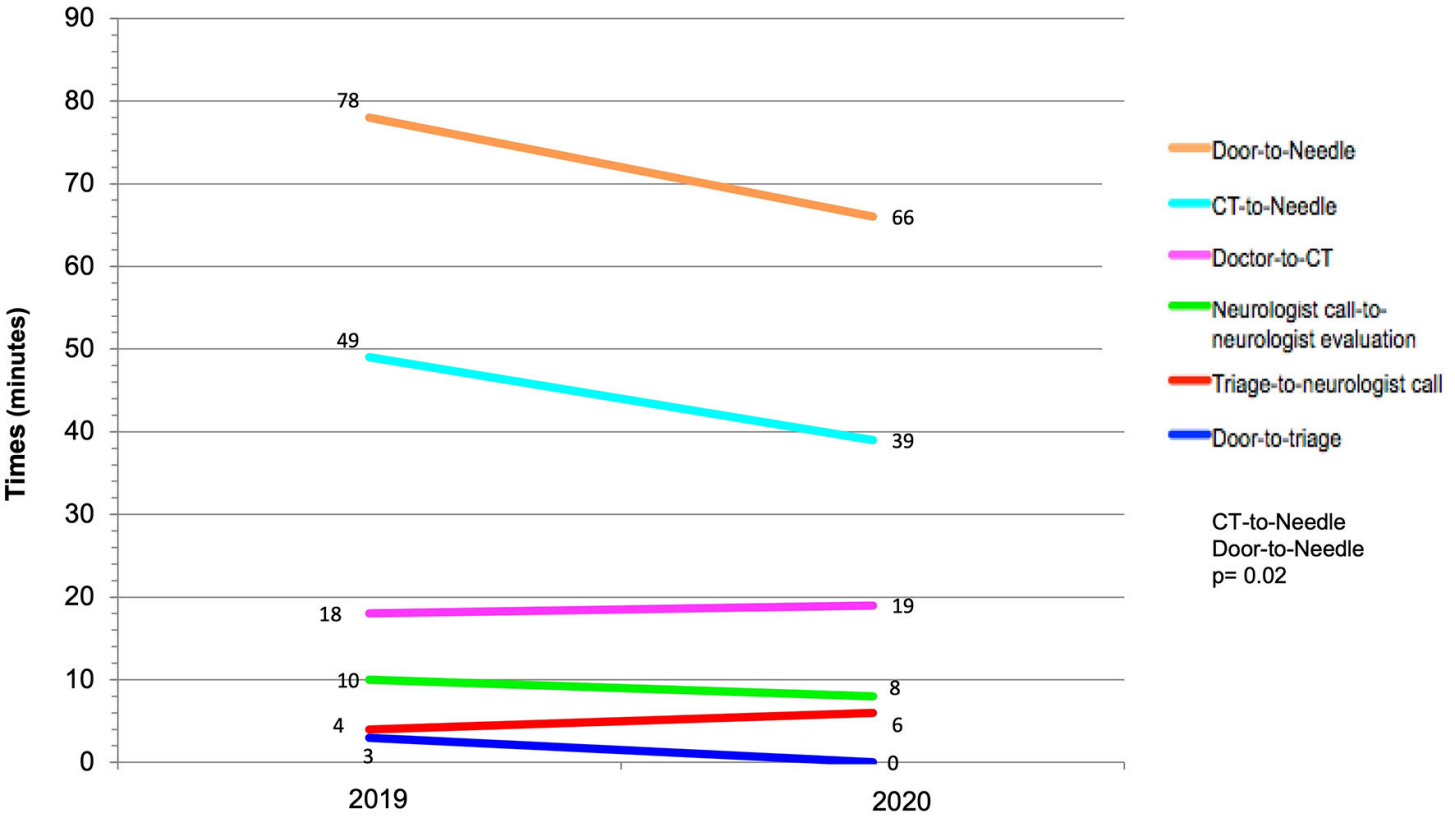
Median time for different quality indicators in acute ischemic stroke care during 2019–2020.

Figure 2 shows the times in each stage of treatment in 2019 and 2020. The door-to-CT scan time of ≤ 25 min was not different in 2019 compared to 2020 (50 vs. 54%, *p* = 0.63). However, CT-to-needle time decreased from 49 to 39 min (*p* = 0.02), and DNT decreased from 78 to 66 min (*p* = 0.02), respectively. The proportion of patients with DNT ≤ 60 min increased significantly (21% in 2019 compared to 44.3% in 2020).

### Effects of the COVID-19 pandemic

The COVID-19 pandemic was an atypical situation that had an impact on stroke care in 2020. The proportion of patients arriving from SAMU decreased from 74% in 2019 to 61% in 2020 (*p* = 0.08). Breaking expectations, the DNT was lower after the start of the pandemic (75 min before vs. 63 min after in 2020, *p* = 0.018), maintaining the decrease that had been occurring since the implementation of the actions (57 min in 2021, 59% of treated patients).

**Figure 3:**
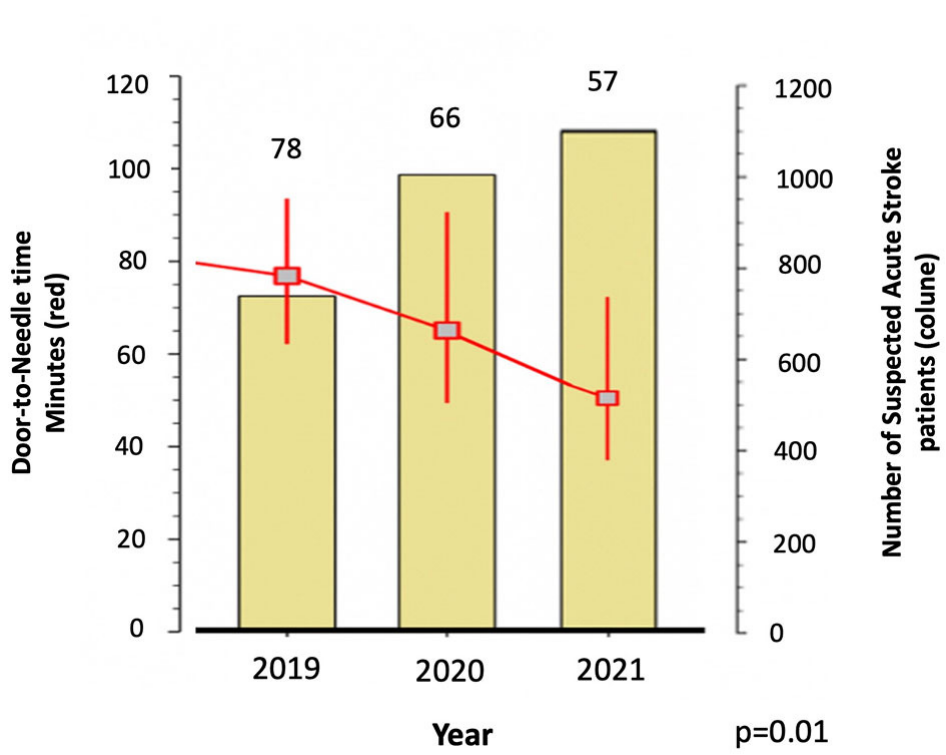
Number of suspected acute stroke patients assisted in the emergency room and door-to-needle-time in each year from 2019 (pre-COVID-19 pandemic) and 2020–2021 (during the COVID-19 pandemic).

In January, 83 patients with acute stroke arrived at the ED, followed by 66 in February, 80 in March, and 44 in April (a reduction of approximately 50% in the first month after the pandemic reached Brazil). Since then, the numbers have recovered, with 1,002 patients with suspected stroke evaluated in 2020 and 1,038 in 2021. More patients arrived out of the 4.5 h window for IVT (35% from January to March and 20% from April to December 2020). The IVT eligibility (IVT treated/all AIS ratio) decreased from 14.5% in 2019 to 8.8% in 2020, rising again to 14.3% in 2021. Patients with AIS treated with IVT after the pandemic were younger (65 years [IQR 59–68] in 2020 vs. 71 years [67–73] in 2019, *p* = 0.011). Figure 3 shows the volume of patients with suspected stroke and DNT in each year from 2019 to 2021, demonstrating that despite the pandemic, it was possible to reduce the metrics for acute stroke care.

## Discussion

Detecting the reasons for the delay in treatment and implementing actions to reduce treatment times are essential to improve patients' functional outcomes (24), reducing disability and stroke mortality even in the most severe cases of large vessel occlusions (25). The study demonstrated the main reasons for the delay in acute stroke treatment in a public university hospital, including during the COVID-19 pandemic, which is a high-volume stroke center. Despite many years of experience, education, frequent training of staff, continuous monitoring of data, and review of service times, this study showed that there are still barriers to overcome in order to reduce treatment times and improve quality.

In general, the median door-to-CT time was 25 min, still within the international standard (9). A door-to-CT time >20 min is considered above our institutional goal to improve treatment times. The median DNT was 73 min despite constant efforts to reduce this time. Although the recommended time of ≤ 60 min is a challenge in most centers, several studies have shown that a median DNT of 20–35 min is possible (6, 8, 11, 26, 27).

The need to perform a brain MRI in AIS of unknown onset symptoms delays treatment by 25 min. Although without statistically significant difference, probably due to the small number of cases, only one of the nine patients selected by MRI was treated in < 60 min (28). Without this exam, however, these patients would not have received reperfusion therapy with IVT. MRI better visualizes the ischemic area in the acute phase compared to CT scan (29), but it is less available, requires more time to perform, and, in the case of this public hospital, the radiology technician is on-call at night, which delays the MRI and consequently the treatment. For these reasons, as a head CT scan is still the gold standard for assessing acute stroke, it is the first option at HCPA.

Patients with a previous history of stroke had a 15-min increase in DNT possibly due to uncertainties about the treatment of a patient with previous functional impairment. Approximately 69% of patients arrived through SAMU. As previously demonstrated in the literature, patients arriving by EMS have a greater chance of receiving IVT and have a lower DNT (6, 8, 10, 11). In our study, patients who arrived by SAMU had an almost 2-fold chance to receive IVT in ≤ 60 min (66% arrived by SAMU vs. 34% from other transportation).

The incorporation of a stopwatch into the stroke protocol has been a standard practice since the hospital was designated as a stroke center. The use of a stopwatch in acute stroke care plays a crucial role in ensuring timely treatment and improving patient outcomes. By accurately measuring the time intervals involved in acute stroke management, a stopwatch provides valuable feedback that enables healthcare professionals to promptly identify and address potential delays. This real-time feedback serves as a powerful tool in identifying areas for improvement. Furthermore, the presence of a visible timekeeping device serves as a constant reminder of the urgency of stroke care, promoting a culture of time sensitivity and adherence to evidence-based guidelines (9, 30–33).

Some factors previously identified in the hospital as barriers that delayed patient care were modified during this study. A critical factor was the distance between the ED and the radiology service and, in addition, the waiting time for the elevator to arrive because the CT scan is located on the second floor. Part of this problem was reduced by IVT infusion on the CT scan table, which was frequently performed in this study. Patients who received treatment on the CT scan table had a median DNT of 52 [IQR 39–71], which is 22 min lower than patients who returned to start IVT at the ASU. Of patients treated on the CT scan table, 67% had a DNT ≤ 60 min compared to 24% of those treated in the ED. Other studies demonstrate that the IVT treatment on the CT scan table is one of the most important factors in reducing the time to treatment (6, 8, 10, 11).

Diabetic patients received IVT in < 60 min more often than non-diabetics. One possible explanation for this finding is that patients with diabetes may have more severe strokes, which would require prompt initiation of treatment. It is also possible that healthcare providers may more closely monitor and manage diabetic patients, leading to faster identification, and treatment of stroke symptoms. In general, this is an interesting observation that requires further investigation. Some studies show that patients with higher NIHSS tend to have a shorter DNT (34). In our study, patients with NIHSS ≥ 15 had a tendency to have a higher median DNT (81 vs. 69) but without statistical significance (*p* = 0.095). In the multivariate analysis, the independent factors for treatment delay were previous AF (OR 7.0) and IVT in the ED (OR 9.0). Patients with previous AF frequently have more severe strokes and need to be assessed for the use of anticoagulants, and some of them need to wait for the INR result in the case of vitamin K antagonist use. Additionally, AF patients may have a higher risk of bleeding complications during thrombolysis treatment, which can also impact the decision-making process and delay the treatment. Regarding IVT in the ED, probably the time to move the patient out of the CT scan room, return to the ED, and place the patient on a bed in the stroke unit is an important delay factor, which can be reduced by treating patients on the CT scan table.

In the evaluation of all stages of the protocol until the start of IVT, the most frequent delay factor in treatment (considering that each patient could have more than one reason for delay) was the time between the CT scan and the start of IVT treatment. Several factors may be associated with treatment delay after a CT scan without contrast: the need to perform CT angiography (35, 36), delay in lowering blood pressure (27), difficult cases and decision-making delay, lack of information about the patient, and the need to wait for a family member, and delayed waiting for INR results in anticoagulated patients due to lack of point-of-care INR testing in the hospital. Approximately 90% of patients had a CT-to-treatment >20 min. The second most frequent reason was IVT in the ED, followed by a delay in performing the CT scan and CT angiography. Removing the treatment site as a treatment delay cause since the treatment on the CT scan table was implemented during the study, we consider the single most important reason for the delay in treatment in each patient was (1) the delay to perform the CT scan in 22% of cases, followed by (2) a delay in starting treatment after CT scan due to delay in the decision-making process in 19%, and (3) a delay in reducing blood pressure in 11% (Table 5). All these reasons have been described as causes of treatment delay in the international literature (6, 8, 10, 11, 26–29, 34). Only 8% of patients showed no delay in any of the stages, demonstrating that there are still many opportunities to reduce treatment times.

Several actions implemented during the study period certainly contributed to the reduction of DNT between the evaluated period, from 78 to 66 min, with a reduction of the CT-To-needle time from 49 to 39 min. The main factor to decrease the DNT was performing IVT bolus and starting the continuous infusion on the CT scan table, especially in patients who started the bolus on the CT scan table before the CT angiography acquisition. Despite the door-to-CT being the main isolated cause of delay, the median time was 25 min and remained between 2019 and 2020.

Despite all attempts to decrease stroke care times, some centers have enormous success leading to media DNT between 20 and 30 min; however, in most centers, it is still difficult to reach DNT ≤ 60 min. The quality program of the American Stroke Association (Get with the Guidelines) demonstrates that the program helps to progressively decrease these times, but are still not ideal, with the majority of American hospitals presenting a median DNT of >60 min (37).

The COVID-19 pandemic, as in other parts of the world, brought an initial drop in the number of stroke patients arriving at the hospital. In addition, there was a significant drop in eligibility for thrombolysis and in the number of patients treated (reduction from 35% of patients arriving within 4.5 h before the pandemic to 20% after). The initial decrease in cases was followed by an increase in the number of stroke patients. It is possible that the increase in stroke admissions at our center during the pandemic was due to the fact that our center is a reference for a population with a higher prevalence of stroke risk factors, including hypertension, and diabetes. Additionally, the implementation of policies and protocols to ensure timely and appropriate care for stroke patients during the pandemic may have contributed to this increase.

Acute stroke protocols have been adjusted according to national indications for protection against COVID-19 infection. As all stroke patients were tested for COVID-19 infection and evaluated with appropriate personal protective equipment, it was not necessary to wait for COVID test results to proceed with acute stroke reperfusion treatment. Many patients received IV thrombolysis bolus and started continuous infusion of tPA in the radiology department before returning to the ED. DNT continued to decline in 2021, even during the pandemic, with the implementation of the new measures despite the greater number of patients arriving with AIS in this period.

Our study has limitations. The study was conducted at a single hospital, which may limit the generalizability of the findings to other healthcare settings. The study covered the period from 2019 to 2021, which was affected by the COVID-19 pandemic. The improvement in DNT observed in 2020 compared to 2019 could be attributed to the less satisfactory DNT in the previous year. Additionally, the study focused on treatment times and did not assess long-term functional outcomes. Therefore, the impact of treatment times on patient outcomes was not evaluated.

## Conclusion

The monthly volumes for IVT and stroke hospitalizations reduced at the beginning of the COVID-19 pandemic in our center, but the delivery of acute stroke care remained a priority. Continuing education and staff training help to reduce IVT times for stroke patients despite all necessary safety measures during acute stroke care due to the risk of coronavirus infection. In this study, the main reason for treatment delay was the delay in performing CT scan, followed by the decision-making process delay. The implementation of the IVT bolus and the start of continuous infusion on the CT scan table was the main factor in reducing DNT during the COVID-19 pandemic. Monitoring data in a public hospital in a middle-income country allows us to identify the main reasons for treatment delay and to plan specific actions. New measures to be implemented soon will help to reduce even more the treatment times: CT scan in the ED and the use of point-of-care INR testing for anticoagulated patients.

## Data availability statement

The raw data supporting the conclusions of this article will be made available by the authors, without undue reservation.

## Ethics statement

The studies involving human participants were reviewed and approved by Comitê de Ética em Pesquisa do Hospital de Clínicas de Porto Alegre. The Ethics Committee waived the requirement of written informed consent for participation.

## Author contributions

SM designed the study, analyzed the data, and wrote the first draft and the final version of the document. MK collected the data, wrote and reviewed the first draft, and approved the final version. AS and LC wrote the first draft of the document. TS wrote the final version of the document. All others collected the data and reviewed the final version of the document.
